# High expression of AFAP1-AS1 is associated with poor prognosis of digestive system cancers: A meta-analysis

**DOI:** 10.1097/MD.0000000000030833

**Published:** 2022-09-23

**Authors:** Xiaona Xu, Fujiao Duan, Liran Xu, Shiutin Ng, Yongwei Li, Yanan Li, Xiaoge Wang, Tianjian Long, Nana Ding, Erping Xu

**Affiliations:** a School of Traditional Chinese Medicine (Zhongjing College), Henan University of Traditional Chinese Medicine, Zhengzhou, China; b Laboratory of Molecular Pathology and Medicine, Zhengzhou University Tumor Hospital, Zhengzhou, China; c The First Affiliated Hospital of Henan University of Traditional Chinese Medicine, Henan Provincial Key Laboratory of Traditional Chinese Medicine Prevention and Treatment of Viral Diseases, Zhengzhou, China; d The First Clinical Medical College of Henan University of Traditional Chinese Medicine, Zhengzhou, China; e Department of Laboratory Medicine, Henan Provincial Hospital of Traditional Chinese Medicine, Zhengzhou, China.

**Keywords:** AFAP1-AS1, DSC, expression, long non-coding RNA, meta-analysis, prognosis

## Abstract

Actin filament-associated protein 1 antisense RNA 1 (AFAP1-AS1) is associated with prognosis in many cancers. The aim of this study was to systematically evaluate the potential correlation between AFAP1-AS1 and the prognosis of digestive system cancers (DSC).

**Methods:**

EMBASE, Web of Science, Cochrane Library, PubMed, Wanfang Data (Chinese), and CNKI (Chinese) were comprehensively searched for literature published from the establishment of the database to September 2021.All case-control studies that met the inclusion criteria were retrieved; additionally manual retrieval and literature tracing was performed. After extracting the relevant data, Revman 5.3.5 software was used for meta-analysis.

**Results:**

Eighteen studies were included in analyses, high expression of AFAP1-AS1 was significantly correlated with poor prognosis in DSC, including overall survival (HR = 1.93, 95% CI: 1.72–2.17, *P* < .001) and disease-free survival/progression-free survival (HR = 1.87, 95% CI: 1.56–2.26, *P* < .001). In addition, the expression of AFAP1-AS1 was significantly correlated with tumor size, tumor stage, and lymph node metastasis.

**Conclusion:**

High expression of AFAP1-AS1 was associated with poor prognosis in DSC. Therefore, it could be used as a potential marker for evaluating prognosis in DSC

## 1. Introduction

Digestive system cancers (DSC) are among the most common cancers worldwide.^[1]^ The 2020 data released by the International Agency for Research on Cancer (IARC)^[2]^ showed that new cases and deaths of major DSC (esophageal, gastric, hepatic, colorectal, pancreatic, and gallbladder cancers) accounted for 26.65% and 36.44% of all cancers, respectively, and showed an increasing trend.^[3]^ Many cases of DSC are occult,^[4]^ and most of them develop to the middle and late stages. DSC prognosis is poor, and there are no known early diagnostic markers. Researchers have been actively investigating potential markers of DSC.^[5]^

Actin filament-associated protein 1 antisense RNA 1 (AFAP1-AS1) (6810 nucleotides) is located on chromosome 4 of human genes. It is involved in a variety of physiological functions and cancer behaviors, such as migration, invasion, metastasis, and angiogenesis.^[6–9]^ In recent years, AFAP1-AS1 has been found to be abnormally expressed in many cancers, including cholangiocarcinoma, pancreatic adenocarcinoma, hepatocellular carcinoma,^[6–8]^ colorectal cancer, gastric cancer, and esophageal cancer.^[10–12]^

At present, many studies on AFAP1-AS1 and cancers are ongoing; however, the number of cases reported in each is limited. The specific target of long non-coding RNA (lncRNA) AFAP1-AS1 and its role in the occurrence or prognosis of DSC are still unclear, and further research is needed. Thus, we conducted a meta-analysis to determine the prognostic significance of AFAP1-AS1 expression in DSC.

## 2. Materials and Methods

The present study was performed according to Preferred Reporting Items for Systematic Reviews and Meta-Analyses (PRISMA) statement and the Meta-analysis of Observational Studies in Epidemiology (MOOSE). Owing to its design, the study did not require ethical approval or informed consent.

## 3. Search strategy

The system retrieved literature published from the establishment of the database to September 15, 2021. The databases include Embase, Web of Science, Cochrane Library, PubMed, Wanfang Data (Chinese), and CNKI (Chinese). The search terms were “cancer,” “long non-coding RNA AFAP1-AS1/LncRNA AFAP1-AS1,” and “survival/prognosis.” In addition, the relevant reference articles not identified through database retrieval were manually searched to avoid potential omissions.

### 3.1. Inclusion and exclusion criteria

The inclusion criteria were as follows: the expression level of AFAP1-AS1 detected in DSC; correlation analysis between AFAP1-AS1 expression and overall survival (OS), disease-free survival/progression-free survival (DFS/PFS), or other survival indicators; patients with cancer divided into high expression group and low expression group; hazard ratio (HR) and 95% confidence interval (95% CI) provided or calculated indirectly from the survival curve; and the research object included tissue.

Exclusion criteria were as follows: the type of article was a review, meta-analysis, letter, case report, or expert opinion; 95% CI and HR could not be obtained; the samples collected from subjects were cells, stool, or serum; and duplicate literature.

### 3.2. Data extraction

The identified articles were independently evaluated by 2 authors (X.X. and X.W.), and disagreements were resolved by the third author (F.D.). The following information was extracted from the articles: first author, year of publication, country, follow-up time, sample size, cancer type and survival analysis results (univariate and/or multivariate analysis), HR, and 95% CI. All data were analyzed as independent datasets. If HRs and 95% CIs were not provided in the original text, Kaplan–Meier (KM) curves were extracted and estimated using methods published by Parmar et al^[13]^ and Tierney et al.^[14]^

### 3.3. Quality assessment

The Newcastle–Ottawa Scale (NOS) was used to evaluate the quality of the literature included in the study, which included 3 categories (selection, comparability, and exposure), a total of 8 items, with a full score of 9. Studies with a score ≥ 7 were considered to be of high quality. The quality of prognostic studies was evaluated according to the method by Hayden et al^[15]^ for assessing potential biases, which included study participation, study attrition, prognostic factor measurement, outcome measurement, study confounding, statistical analysis, and reporting.

### 3.4. Statistical analysis

The combined HRs with 95% CIs were conducted using Review Manager 5.3.5 (Cochrane Collaboration, Oxford, UK) to evaluate the relationship between AFAP1-AS1 expression level and prognosis. Inter-study heterogeneity index was tested using *Q* tests and *I*^2^.^[16]^ According to the results of heterogeneity analysis, when the *P* heterogeneity value was ≥0.1 or *I*^2^ ≤ 50%, the fixed effects model (Mantel–Haenszel method)^[17]^ was applied to calculate the combined effect size, otherwise (*P* < .1 or *I*^2^ > 50%), the random effects model (DerSimonian and Laird method) was used.^[18]^

For articles that did not provide HR, 95% CI, or *P* values, the Engauge Digitizer 10.0 (https://sourceforge.net/projects/digitizer/) was used to extract the original survival data from the KM curve. Subgroup analysis was performed for different types of cancer, and publication bias was detected using Begg^[19]^ and Egger^[20]^ tests.

If the 95% CI did not contain the value 1 and the combined HR > 1, the finding was considered to be statistically significant. All *P* values were 2-sided, and statistical significance was set at *P* < .05. A sensitivity analysis was performed to determine the reliability of the combined results. The combined HRs of 95% CIs were calculated to evaluate the correlation between AFAP1-AS1 expression and clinicopathological factors. Statistical significance was set at *P* < .05.

## 4. Results

### 4.1. Literature search and characteristics of eligible studies

**Figure 1.**
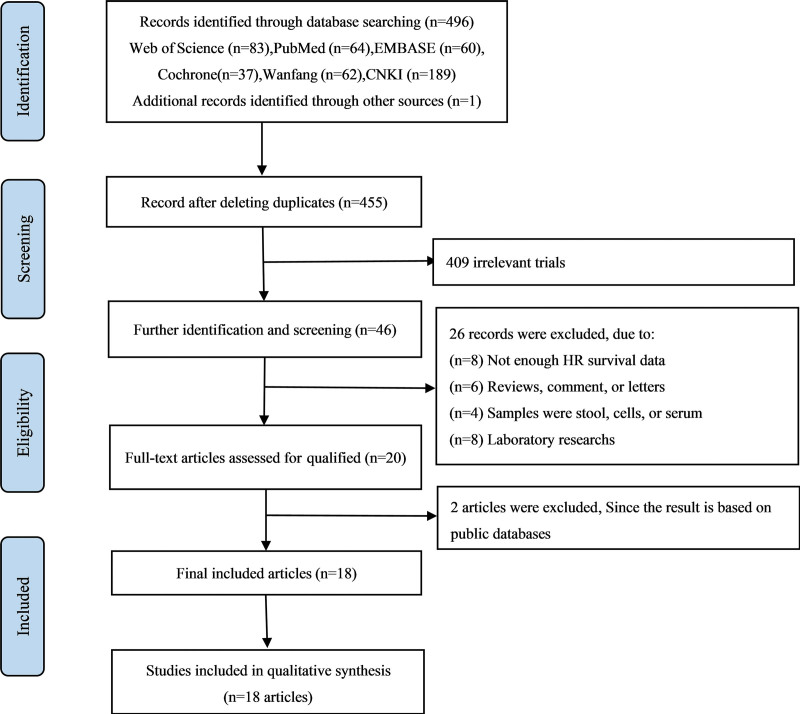
Flow chart of literature retrieval and research selection.

Figure 1 shows a flowchart of the retrieval process. A total of 496 studies were retrieved. First, 41 duplicate articles were excluded, and then 409 non-DSC articles were excluded. Due to incomplete data, article type (conference papers, reviews), non-tissue samples, mechanism research, and other reasons, 20 articles were excluded; another 2 articles based on database search were excluded. Finally, 18 eligible studies were included.^[6,21–37]^

### 4.2. Baseline characteristics of the included studies

Table 1 shows the characteristics of the included studies. The studies were published between 2015 and 2021 and included 1440 patients with OS data and 572 patients with DFS/PFS data. The studies covered gastric cancer, colorectal cancer, cholangiocarcinoma, pancreatic adenocarcinoma (PAAD), hepatocellular carcinoma (HCC), esophageal squamous cell carcinoma (ESCC), and gallbladder carcinoma (GBC), and all used real-time fluorescent quantitative polymerase chain reaction (qRT-PCR) to detect of AFAP1-AS1. The specimens were frozen tissues, and the critical value of AFAP1-AS1 expression was median or normal.

**Table 1:** Basic characteristics of included studies.

Author	Year	Country	Ethnicity	Number		Histology	TNM stage	Sample	Assay	Follow-up (mo)	Cutoff	Analysis	Outcome
				OS	DFS/PFS								
Zhao et al^[21]^	2017	China	Asian	80		Gastric cancer	I–IV	Frozen tissue	qRT-PCR	40	Normal	KM	SC
Feng et al^[22]^	2018	China	Asian	91		Gastric cancer	I–IV	Frozen tissue	qRT-PCR	70	Median	KM/CR	HR/SC
Qiao et al^[23]^	2017	China	Asian	87		Gastric cancer	I–IV	Frozen tissue	qRT-PCR	60	Median	KM	SC
Dang et al^[24]^	2021	China	Asian	97		Gastric cancer	I–IV	Frozen tissue	qRT-PCR	120	Normal	KM	HR/SC
Ye et al^[25]^	2018	China	Asian	66		Gastric cancer	I–IV	Frozen tissue	qRT-PCR	26	Median	CR	HR
Ma et al^[26]^	2020	China	Asian	80		Gastric cancer	I–IV	Frozen tissue	qRT-PCR	60	Median	KM	SC
Li et al^[27]^	2016	China	Asian	30		Colorectal cancer	II–III	Frozen tissue	qRT-PCR	30	Normal	KM	SC
Wang et al^[28]^	2016	China	Asian	52	DFS,52	Colorectal cancer	I–IV	Frozen tissue	qRT-PCR	50	Median	KM/CR	HR/SC
Li et al^[29]^	2018	China	Asian	56	DFS,56	Colorectal cancer	I–IV	Frozen tissue	qRT-PCR	60	Normal	KM	SC
Tang et al^[30]^	2018	China	Asian	80		Colorectal cancer	NA	Frozen tissue	qRT-PCR	100	Normal	KM	SC
Ye et al^[31]^	2015	China	Asian	90	PFS,90	PAAD	TN	Frozen tissue	qRT-PCR	60	Median	KM	SC
Fu et al^[32]^	2016	China	Asian	80		PAAD	I–IV	Frozen tissue	qRT-PCR	50	Median	KM	SC
Chen et al^[33]^	2018	China	Asian	63		PAAD	I–IV	Frozen tissue	qRT-PCR	60	Normal	KM	SC
Lu et al^[34]^	2016	China	Asian	156	DFS,156	HCC	I–Ⅲ	Frozen tissue	qRT-PCR	80	Median	KM	SC
Zhang et al^[35]^	2016	China	Asian	78		HCC	I–Ⅳ	Frozen tissue	qRT-PCR	60	Normal	KM/CR	HR/SC
Zhou et al^[36]^	2016	China	Asian	162	PFS,162	ESCC	I–Ⅳ	Frozen tissue	qRT-PCR	80	Median	KM/CR	HR/SC
Lu et al^[6]^	2017	China	Asian	56		Cholangiocarcinoma	I–Ⅲ	Frozen tissue	qRT-PCR	80	Median	KM	SC
Ma et al^[37]^	2016	China	Asian	40		GBC	I–Ⅳ	Frozen tissue	qRT-PCR	40	Median	KM	SC

CR = Cox regression, DFS = disease-free survival, ESCC = esophageal squamous cell carcinoma, GBC = gallbladder carcinoma, HCC = hepatocellular carcinoma, HR = hazard ratio, KM = Kaplan–Meier, OS = overall survival, PAAD = pancreatic adenocarcinoma, PFS = progression-free survival, qRT-PCR = quantitative real-time PCR, SC = survival curve, TNM = tumor-node-metastasis.

### 4.3. Quality assessment

The quality assessment according to the Quality in Progress Studies (QUIPS) tool is summarized in Table 2. The NOS scores of eligible articles ranged from 5 to 9 (Table S1, Supplemental Digital Content, http://links.lww.com/MD/H407), with an average of 6.78, and 83.3% (15/18) were considered high-quality studies.

**Table 2:** Quality assessment of included studies based on QUIPS.

	Quality evaluation of prognosis study						Total score^*^	Level of evidence^†^
Study	Study participation	Study attrition	Prognostic factor measurement	Outcome measurement	Study confounding	Statistical analysis and reporting		
Zhao et al^[21]^	Yes	Partly	Yes	Partly	Partly	Partly	6	2b
Feng et al^[22]^	Yes	Partly	Yes	Yes	Partly	Yes	8	2b
Qiao et al^[23]^	Yes	Partly	Yes	Partly	Partly	Partly	5	2b
Dang et al^[24]^	Yes	Partly	Yes	Partly	Partly	Yes	8	2b
Ye et al^[25]^	Yes	Partly	Yes	Partly	Partly	Partly	6	2b
Ma et al^[26]^	Yes	Partly	Yes	Partly	Partly	Partly	7	2b
Li et al^[27]^	Partly	Yes	Yes	Partly	Partly	Partly	7	2b
Wang et al^[28]^	Yes	Partly	Yes	Yes	Partly	Yes	8	1b
Li et al^[29]^	Yes	Yes	Yes	Partly	Partly	Partly	6	2b
Tang et al^[30]^	Yes	Partly	Yes	Partly	Partly	Partly	7	2b
Ye et al^[31]^	Yes	Partly	Yes	Partly	Partly	Partly	5	2b
Fu et al^[32]^	Yes	Partly	Yes	Partly	Partly	Partly	7	2b
Chen et al^[33]^	Yes	Partly	Yes	Partly	Partly	Partly	7	2b
Lu et al^[34]^	Yes	Partly	Yes	Partly	Partly	Partly	5	2b
Zhang et al^[35]^	Yes	Partly	Yes	Yes	Partly	Yes	8	2b
Zhou et al^[36]^	Yes	Yes	Yes	Yes	Partly	Yes	9	1b
Lu et al^[6]^	Yes	Partly	Yes	Partly	Partly	Partly	7	2b
Ma et al^[37]^	Yes	Partly	Yes	Partly	Partly	Partly	6	2b

QUIPS = Quality in Progress Studies.

* Quality assessment of included studies based on the Newcastle–Ottawa Scale.

† The level of evidence was estimated for all included studies with the Oxford Centre for Evidence Based Medicine criteria. QUIPS = Quality in Progress Studies.

### 4.4. Expression of AFAP1-AS1 in DSC tissue

**Figure 2.**
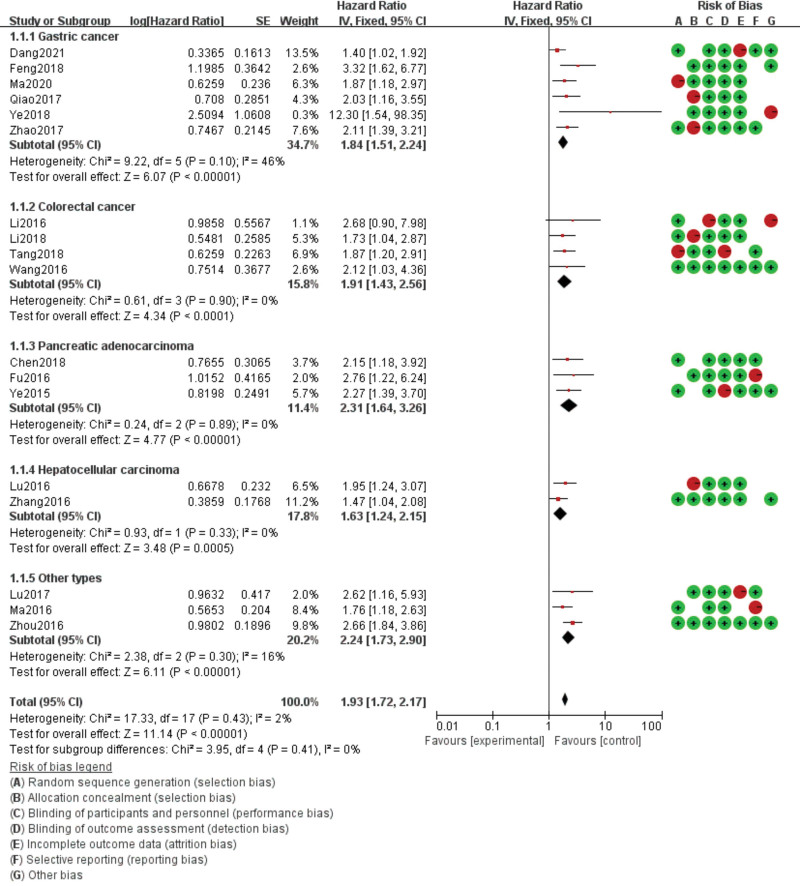
Forest diagram of the relationship between AFAP1-AS1 expression and overall survival (OS). AFAP1-AS1 = Actin filament-associated protein 1 antisense RNA 1.

**Figure 3.**
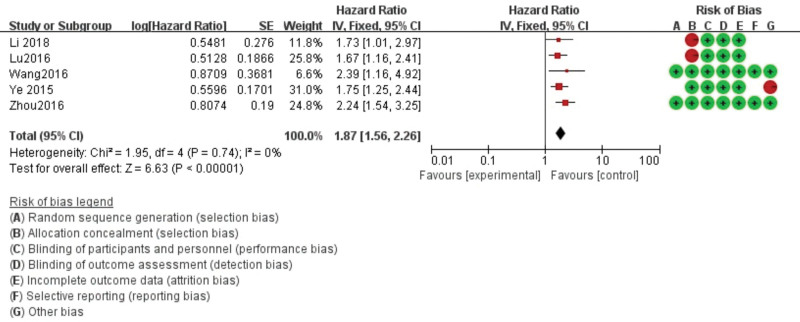
Forest diagram of the relationship between AFAP1-AS1 expression and disease-free survival/progression-free survival (DFS/PFS). AFAP1-AS1 = Actin filament-associated protein 1 antisense RNA 1.

There was no heterogeneity between AFAP1-AS1 and OS (*P* heterogeneity = 0.43 and *I*^2 ^= 2%). Based on these preconditions, the combined HR of AFAP1-AS1 was calculated using the fixed effect. The summary analysis of 18 studies showed that the expression of AFAP1-AS1 was related to OS (HR = 1.93, 95% CI: 1.72–2.17, *P* < .001; Figure 2) and DFS/PFS (HR = 1.87, 95% CI: 1.56–2.26, *P* < .001; Figure 3).

We performed a subgroup analysis by cancer type. The results showed a significant correlation between the high expression of AFAP1-AS1 and OS in gastric cancer (HR = 1.84, 95% CI: 1.51–2.24, *P* < .001), colorectal cancer (HR = 1.91, 95% CI: 1.43–2.56, *P* < .001), PAAD (HR = 2.31, 95% CI: 1.64–3.26, *P* < .001), HCC (HR = 1.63, 95% CI: 1.24–2.15, *P* < .001), and other types of DSC (HR = 2.24, 95% CI: 1.73–2.90, *P* < .001; Figure 2).

### 4.5. Relationship between high expression of AFAP1-AS1 and clinicopathological factors

High expression of AFAP1-AS1 was related to size (>5 cm; HR = 2.23, 95% CI: 1.57–3.16, *P* < .001), degree of differentiation (poor; HR = 1.42, 95% CI: 1.05–1.92, *P* = .02), stage (III, IV; HR = 2.88, 95% CI: 2.18–3.80, *P* < .001), lymph node metastasis (HR = 3.08, 95% CI: 2.18–4.34, *P* < .001), and high tumor-node-metastasis (TNM) stage (HR = 2.11, 95% CI: 1.18–3.77, *P* = .01). There was no correlation with age and sex (Table 3).

**Table 3:** Relationship between expression of AFAP1-AS1 in DSC and clinicopathological factors.

Factors	Number of studies	Number of patients	Pooled HR (95% CI)	*P* value	Heterogeneity		
					*I*^2^ (%)	*P* value	Model
Age (old vs young)	14	1107	1.15 (0.88–1.51)	.31	3.0	.41	Fixed
Gender (male vs female)	14	1107	1.28 (0.97–1.68)	.08	0.0	.97	Fixed
Tumor size (≥5 vs <5 cm)	7	635	2.23 (1.57–3.16)	<.001	14.0	.32	Fixed
Tumor grade (PD vs MD WD)	10	787	1.42 (1.05–1.92)	.02	11.0	.34	Fixed
Tumor stage (III IV vs I II)	13	1017	2.88 (2.18–3.80)	<.001	9.0	.35	Fixed
Lymph node metastasis (present vs absent)	7	617	3.08 (2.18–4.34)	<.001	0.0	.44	Fixed
TNM stage (high vs low)	7	613	2.11 (1.18–3.77)	.01	55.0	.04	Random

CI = confidence interval, DSC = digestive system cancers, MD = moderately-differentiated, OR = odds ratio, PD = poorly-differentiated, TNM = tumor-node-metastasis, WD = well-differentiated.

### 4.6. Sensitivity analysis

**Figure 4.**
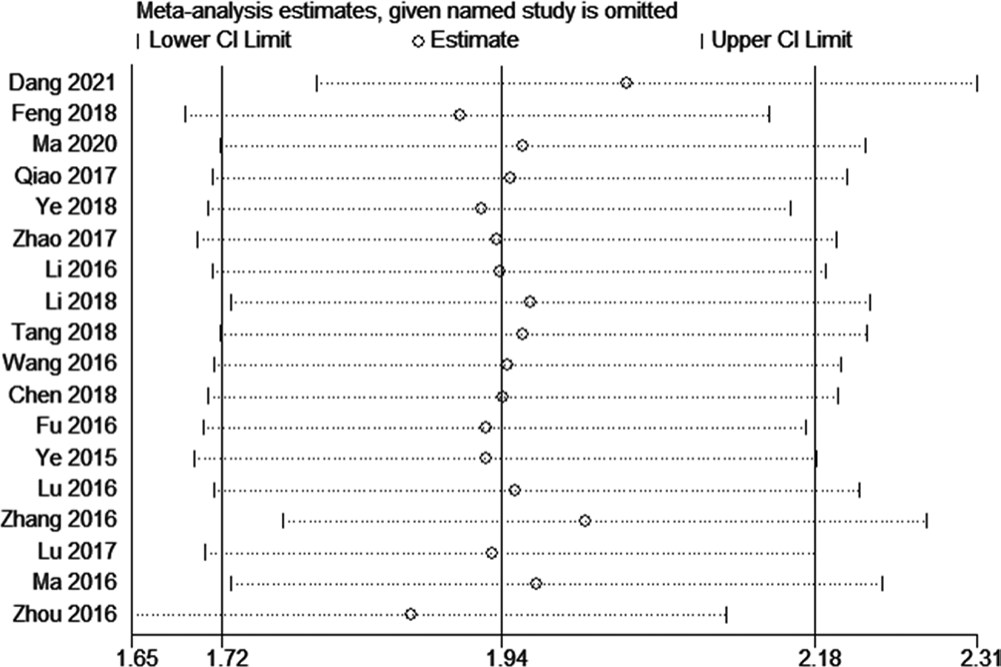
Sensitivity analysis of the association between AFAP1-AS1 expression and overall survival (OS). AFAP1-AS1 = Actin filament-associated protein 1 antisense RNA 1.

To verify the stability of the results, a sensitivity analysis was performed by removing 1 study at a time and recalculating the combined HR. There was no significant change in the results, indicating that our results were reliable (Fig. 4).

### 74.. Publication bias

**Figure 5.**
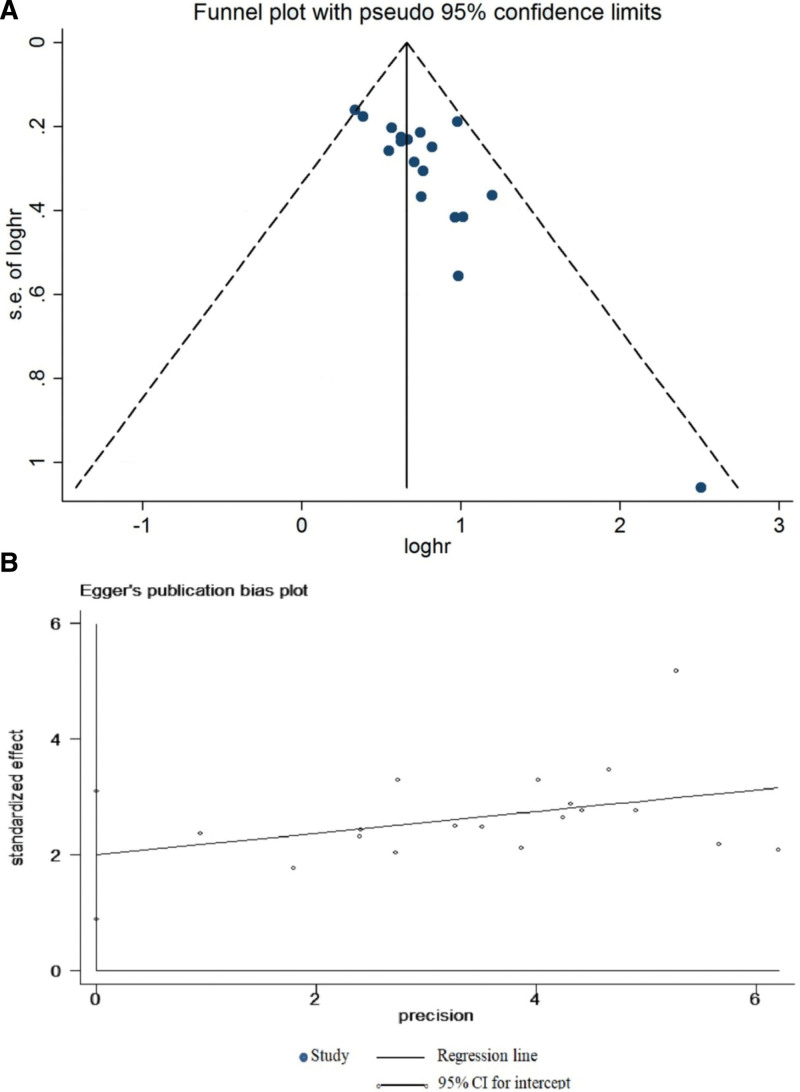
(A) Begg funnel diagram of publication bias on the relationship between AFAP1-AS1 expression and overall survival (OS). (B) Egger funnel diagram of publication bias on the relationship between AFAP1-AS1 expression and OS.

Begg and Egger tests did not show a significant publication bias (Table S2, Supplemental Digital Content, http://links.lww.com/MD/H408). Concurrently, the shape of the funnel diagram was symmetrical (Fig. 5).

## 5. Discussion

LncRNAs are non-coding RNAs with a length of >200 nucleotides, which play an important role in cell proliferation, differentiation, apoptosis, invasion, and immune response.^[38–42]^ Studies have confirmed that lncRNAs participate in oncogenesis^[43,44]^ through epigenetic, transcriptional, and post-transcriptional regulation. Existing diagnostic techniques, such as gastrointestinal endoscopy, can only detect early precancerous lesions and cancers.^[45]^ In recent years, the discovery of the prognostic value of biomolecules has greatly promoted research on lncRNAs. Some of these (such as lncRNA MALAT1) can be used to predict therapeutic effects.^[46]^

In this study, we used multiple online databases to search for studies related to DSC and conduct a quantitative systematic review. The results indicated that AFAP1-AS1 expression was significantly associated with OS. Additionally, we explored the relationship between lncRNA AFAP1-AS1 expression and cancer type and clinicopathological factors in subgroup analysis. These findings indicated that AFAP1-AS1 may be a potential diagnostic and prognostic indicator for DSC. Han et al^[47]^ reported that the combination of AFAP1-AS1 and AUF1 activated the expression of ERBB2 and promoted trastuzumab resistance. Bi et al^[48]^ found that AFAP1-AS1 induced radiation-resistance in 3 negative breast cancers (TNBC) by activating the Wnt/β-catenin signaling pathway. Liu et al^[49]^ reported that AFAP1-AS1 acted on the PI3K/AKT pathway to promote cisplatin resistance in non-small cell lung cancer. It is worth noting that in DSC, AFAP1-AS1 plays multiple roles and affects cancer progression.

AFAP1-AS1, formerly known as afap-110, is an antisense lncRNA, an actin cross-linked protein, and can bind to CSRC. It belongs to the AFAP1, AFAP1 class-1, and AFAP1 like-2/xb-130 family.^[50,51]^ Wu et al^[52]^ was first to report that AFAP1-AS1 was overexpressed in Barrett esophagus and esophageal adenocarcinoma owing to its gene site hypomethylation. After that, Zeng et al^[53]^ analyzed 5 groups of previously published lung cancer gene expression profiles (GEP) in the high-throughput microarray expression profile database. The results showed that AFAP1-AS1 was most significantly expressed in lung cancer, which was related to poor prognosis. Liu et al^[54]^ conducted a meta-analysis pooled from 8 studies. The results indicated that patients with cancer with high expression of AFAP1-AS1 had a higher risk of lymph node metastasis and distant metastasis, and the OS rate, PFS rate, and recurrence free survival (RFS) rate of patients with high expression of AFAP1-AS1 were lower than those with low expression. High expression of AFAP1-AS1 was associated with poor clinical prognosis. Therefore, AFAP1-AS1 may become a potential new biomarker, which could be used to predict the clinical prognosis in cancer. In addition, Luo et al^[55]^ showed that AFAP1-AS1 could up regulate the expression in esophageal squamous cell carcinoma, promote the proliferation of cancer cells, and inhibit their apoptosis. To ensure the reliability and homogeneity of our results, this study was limited to detecting the expression of AFAP1-AS1 in tissues by qRT-PCR. The results revealed that high expression of AFAP1-AS1 may have been an independent adverse prognostic factor. Our study is the first meta-analysis of the relationship between prognosis and AFAP1-AS1 expression in patients with DSC. The study has some limitations. First, not all included studies reported HR. We extracted some HRs and 95% CIs from the survival curves. This calculation method produces some errors. Second, although there is no statistical evidence of publication bias, all eligible studies have been performed in China, which may lead to publication bias. Finally, the truncated value algorithms expressed by AFAP1-AS1 are different, which may lead to errors in the results. Despite these limitations, this study provides important findings on the relationship between AFAP1-AS1 expression and the prognosis of patients with DSC.

## 6. Conclusion

In summary, the high expression of lncRNA AFAP1-AS1 was significantly correlated with poor prognosis in patients with DSC patients. Therefore, it could be used as a potential marker for evaluating prognosis in DSC.

## Acknowledgments

Special thanks go to Professor Xiaofan Lu and Professor Junzi Li for their key guidance in this study.

## Author contributions

**Conceptualization:** Xiaona Xu.

Data curation: Fujiao Duan.

Formal analysis: Liran Xu.

Investigation: Shiutin Ng.

Project administration: Yongwei Li.

Software: Xiaoge Wang.

Supervision: Yanan Li.

Visualization: Nana Ding.

Writing – original draft: Erping Xu, Xiaona Xu.

Writing – review & editing: Tianjian Long, Xiaona Xu.

## Supplementary Material


