# Supplementary material for: High expression of AFAP1-AS1 is associated with poor prognosis of digestive system cancers: A meta-analysis
Source: Medicine (Baltimore). 2022 Sep 23;101(38):e30833. doi: 10.1097/MD.0000000000030833 (PMC9509167; doi:10.1097/MD.0000000000030833)
Supplement: Supplementary file 1 [file medi-101-e30833-s001.pdf]

Supplementary Table 1 Quality assessment of included studies based on the Newcastle–Ottawa scale for assessing the quality of cohort studies

Study	**Selection** (score)				**Comparability** (score)	**Exposure** (score)			
	Representativeness of the exposed cohort	Selection of the non-exposed cohort	Ascertainment of exposure	Outcome of interest was not present at start of study	Based on the design or analysis[a]	Assessment of outcome	Follow-up long enough for outcomes to occur	Adequacy of follow-up of cohorts	Total Score[b]
Zhao et al[21]	1	1	1	1	1	1	0	0	6
Feng et al[22]	1	1	1	1	2	1	1	0	8
Qiao et al[23]	1	0	1	1	0	1	1	0	5
Dang et al[24]	1	1	1	1	2	1	1	0	8
Ye et al[25]	1	1	1	1	1	1	0	0	6
Ma et al[26]	1	1	1	1	1	1	1	0	7
Li et al[27]	1	1	1	1	1	1	0	1	7
Wang et al[28]	1	1	1	1	2	1	1	0	8
Li et al[29]	1	0	1	1	0	1	1	1	6
Tang et al[30]	1	1	1	1	1	1	1	0	7
Ye et al[31]	1	0	1	1	0	1	1	0	5
Fu et al[32]	1	1	1	1	1	1	1	0	7
Chen et al[33]	1	1	1	1	1	1	1	0	7
Lu et al[34]	1	0	1	1	0	1	1	0	5
Zhang et al[35]	1	1	1	1	2	1	1	0	8
Zhou et al[36]	1	1	1	1	2	1	1	1	9
Lu et al[6]	1	1	1	1	1	1	1	0	7
Ma et al[37]	1	1	1	1	1	1	0	0	6

[a]When there was no statistical significance in the response rate between case and control groups by using a chi-squared test ($P > 0.05$), one point was awarded.

[b]Total score was calculated by adding up the points awarded in each item.
