# Supplementary material for: High expression of AFAP1-AS1 is associated with poor prognosis of digestive system cancers: A meta-analysis
Source: Medicine (Baltimore). 2022 Sep 23;101(38):e30833. doi: 10.1097/MD.0000000000030833 (PMC9509167; doi:10.1097/MD.0000000000030833)
Supplement: Supplementary file 2 [file medi-101-e30833-s002.pdf]

Supplementary Table 2 Publication bias of AFAP1-AS1 in Begg's test and Egger's test.

**Comparisons**	**Begg's test**		**Egger's test**		
	z	***p***	***t***	***p***	**95%CI**
OS	2.80	0.005	3.85	0.001	0.899-3.103
DFS/PFS	0.73	0.462	0.67	0.549	-3.333-5.122

Abbreviations: OS, overall survival; DFS, disease-free survival; PFS, progression-free survival
